# Heat Shock Protein 90 regulates encystation in *Entamoeba*

**DOI:** 10.3389/fmicb.2015.01125

**Published:** 2015-10-13

**Authors:** Meetali Singh, Shalini Sharma, Alok Bhattacharya, Utpal Tatu

**Affiliations:** ^1^Department of Biochemistry, Indian Institute of ScienceBangalore, India; ^2^School of Life Sciences, Jawaharlal Nehru UniversityNew Delhi, India

**Keywords:** *Entamoeba*, Hsp90, phagocytosis, encystation, 17-AAG, Cyst

## Abstract

Enteric protozoan *Entamoeba histolytica* is a major cause of debilitating diarrheal infection worldwide with high morbidity and mortality. Even though the clinical burden of this parasite is very high, this infection is categorized as a neglected disease. Parasite is transmitted through feco-oral route and exhibit two distinct stages namely – trophozoites and cysts. Mechanism and regulation of encystation is not clearly understood. Previous studies have established the role of Heat shock protein 90 (Hsp90) in regulating stage transition in various protozoan parasites like *Giardia, Plasmodium, Leishmania*, and *Toxoplasma*. Our study for the first time reports that Hsp90 plays a crucial role in life cycle of *Entamoeba* as well. We identify Hsp90 to be a negative regulator of encystation in *Entamoeba.* We also show that Hsp90 inhibition interferes with the process of phagocytosis in *Entamoeba*. Overall, we show that Hsp90 plays an important role in virulence and transmission of *Entamoeba.*

## Introduction

*Entamoeba histolytica* is a primitive protozoan and causative organism of amoebiasis. It is estimated that around 50 million people are infected with *E. histolytica* in tropical and developing nations. *Entamoeba* infection could either be asymptomatic or present itself as invasive intestinal amoebiasis with symptoms including colitis, dysentery, and toxic megacolon. If not checked in time, the disease could manifest as invasive extra-intestinal amoebiasis including amoebic liver abscess. Fatality rate for amoebiasis is higher compared to giardiasis caused by a related enteric parasite *Giardia.* The frontline drug for treatment of amoebiasis is metronidazole. However, there are many dose-related side effects associated with the drug and there is emergence of drug resistance as well (Freeman et al., 1997).

*Entamoeba* has a biphasic life cycle. The two stages of its life cycle are trophozoite and cyst. Human is the only known host of *E. histolytica.* Infection of the host happens upon ingestion of food contaminated by *Entamoeba* cysts. Cysts are environmentally resistant infective stage of *Entamoeba.* Ingested cysts undergo excystation to form trophozoites in small intestine. Trophozoites are the pathological stage of the life cycle and colonize large intestine, where they rapidly proliferate (Aguilar-Diaz et al., 2011). Virulence of trophozoites is characterized by their ability of phagocytosis. Phagocytosis is a well-studied process in mammalian host defense system. But our understanding of phagocytosis process in *Entamoeba* is still in its nascence. Many studies have identified signaling pathways regulating phagocytosis and proteome of phagosomes has been recently elucidated (Huston et al., 2003; Marion et al., 2005; Okada et al., 2005; Okada and Nozaki, 2006; Tovy et al., 2011; Mansuri et al., 2014).

For development of new therapeutics against *Entamoeba*, it is essential to understand the regulation of its virulence and life cycle. There have been few efforts by different groups to understand the regulation and mechanism of stage transition in *Entamoeba* (Ehrenkaufer et al., 2007, 2013; De Cádiz et al., 2013; Mi-Ichi et al., 2015). However, questions like: what are the cues for stage transition, how stage transition is regulated and what is the mechanism of stage transition; are largely unanswered.

Heat shock protein 90 (Hsp90), a key molecular chaperone, has been implicated to play a crucial role in growth and life cycle of protozoa like *Giardia, Plasmodium, Leishmania, Toxoplasma*, and *Trypanosoma* (Wiesgigl and Clos, 2001; Graefe et al., 2002; Banumathy et al., 2003; Echeverria et al., 2005; Nageshan et al., 2014; Rochani et al., 2014). In *Giardia*, Hsp90 has been shown to regulate encystation and inhibition of Hsp90 promotes encystation (Nageshan et al., 2014). In *Plasmodium falciparum* Hsp90 regulates ring to trophozoites transition (Banumathy et al., 2003). Hsp90 is an abundant protein and regulates various biological pathways. Its clients include transcription factors, kinases and other non-signaling proteins like telomerase etc. (Taipale et al., 2010). Hsp90 is a dimeric protein with three domains. The N-terminal domain is the ATP binding domain and links to the middle domain via a flexible linker. Middle domain interacts with various clients and co-chaperones and also harbors the critical catalytic Arg. C-terminal is responsible for dimer formation (Pearl and Prodromou, 2006; Taipale et al., 2010). Hsp90 function is regulated by many co-chaperones, which are well conserved in higher eukaryotes (Johnson and Brown, 2009).

Many of the signaling proteins identified in phagosome proteome like Rab11, PAK6, TOR, and Rac1 are known interactors of Hsp90 in mammalian cells (Ohji et al., 2006; Keestra et al., 2013; Bozza et al., 2014). Hsp90 has also been shown to regulate phagocytosis in murine macrophage cell lines (Yan et al., 2004).

In our previous study, we had shown that *Entamoeba* Hsp90 is an active ATPase and its activity was inhibited by pharmacological inhibitor of Hsp90 – 17-allylamino-17-demethoxygeldanamycin (17-AAG; Singh et al., 2014). Others and we have also shown that Hsp90 is crucial for survival and growth of *E. histolytica* (Debnath et al., 2014; Singh et al., 2014). Inhibition of Hsp90 by 17-AAG results in death of *Entamoeba* trophozoites. *Entamoeba* also has a minimal co-chaperone repertoire and lacks many conserved co-chaperones (Singh et al., 2014).

In the current study, we have examined specific roles of Hsp90 in *Entamoeba* life cycle. We found Hsp90 to regulate the process of phagocytosis and encystation. We show that pharmacological inhibition of Hsp90 in trophozoites of *E. histolytica* interferes with the process of phagocytosis. Further, we have used *E. invadens* as a model for encystation to examine the role of Hsp90 in stage transition. We have analyzed the transcriptome data available at amoebadb.org and observed that Hsp90 expression levels along with few of its co-chaperones decrease in cysts followed by an increase during excystation. We now provide an experimental evidence for the role of Hsp90 in encystation process using pharmacological approach. We observe an increase in encystation upon Hsp90 inhibition. Together our results provide evidence for the role of Hsp90 in essential processes such as phagocytosis in actively proliferating trophozoites. Inhibition of Hsp90 function with sub lethal doses of Hsp90 inhibitors disrupts growth of trophozoites and promotes encystation.

## Materials and Methods

### Culture

*Entamoeba histolytica* strain HM-1: IMSS was maintained in TYI-S-33 medium at 33.5°C containing 15% heat inactivated adult bovine serum (Himedia), and 2.5% Diamond vitamin mix (Sigma; Diamond et al., 1978). *E. invadens* was maintained in TYI-S-33 medium at 25°C.

### Phagocytosis Assay

To quantify phagocytosis rate of *E. histolytica*, 5 × 10^4^ *Entamoeba* cells were incubated with 10^7^ washed RBCs in TYI-S-33 medium at 37°C for 1 h in 1 mL culture medium. Cells were centrifuged and non-phagocytized RBCs were lysed with cold distilled water and *Entamoeba* cells were harvested by centrifugation at 1000*g* for 2 min. Cells were washed with PBS. Cells containing engulfed RBCs were lysed in 1 mL formic acid and absorbance was recorded at 397 nm (Somlata et al., 2012). Cells were also observed under microscope to ascertain phagocytosis. To test the effect of Hsp90 inhibition on phagocytosis, cells were pre treated with 600 nM 17-AAG for 24 h. 0.2% DMSO was used to treat control cells.

### Immunofluorescence Assay

*Entamoeba histolytica* cells were resuspended in incomplete TYI-S-33 medium and transferred onto acetone-cleaned coverslips placed in a petridish and allowed to adhere for 5 min at 37°C. Cells were fixed with 3.7% paraformaldehyde (PFA) in PBS at 37°C for 30 min after taking out the culture medium. Fixed cells were permeabilized with 0.1% Triton-X-100 in PBS for 3 min and washed with PBS followed by quenching with 50 mM NH_4_Cl in PBS for 30 min. The coverslips were then blocked with 1% BSA in PBS for 1 h and then incubated with primary antibody for 1 h at 37°C. After that, cells were washed with 1% BSA in PBS and labeled with secondary antibody at 37°C for 30 min. Antibody dilutions used are as follows: α-EhHsp90 was used at 1:100, TRITC-phalloidin (Sigma; 1 mg/ml) at 1:200 and anti-rabbit Alexa-488 (Molecular probes) at 1:200. The stained cells were washed with PBS followed by mounting on a glass slide using DABCO {1,4-diazbicyclo (2,2,2) octane} (sigma) as antifade. The edges of coverslips were sealed to avoid drying. Confocal images were taken using an Olympus Fluoview FV1000 laser-scanning microscope.

### Cell Viability Assay

Cell viability assay was carried out as described previously (Singh et al., 2014). Briefly, 15,000 *E. invadens* trophozoites grown to log phase were seeded per well in TYI-S-33 medium or LG medium in a 96-well plate. Cells were treated for 24 h with 17-AAG (concentration varying from 10 nM to 100 μM). DMSO (0.2%) was used as control. Cell viability was assessed by trypan blue dye exclusion. 50% Growth inhibitory concentration (GI_50_) was calculated by plotting percent survival against Log_10_ 17-AAG concentration.

### Encystation

*Entamoeba invadens* trophozoites grown to log phase were chilled on ice for 5 min and harvested by centrifugation at 600g for 5 min. Trophozoites were induced for encystation in LG medium (TYI medium without glucose diluted to 47% with 5% adult bovine serum and 2.5% Diamond vitamin mix) at a concentration of 5 × 10^5^ cells/mL (Singh et al., 2011). Induction was carried out for 72 h. Cysts were identified under microscope by their spherical refractile morphology and staining of chitin cell wall by caulcoflour white. Number of cysts formed, was scored by counting cysts resistant to detergent (0.05% SDS). For effect of Hsp90 inhibition on encystations, trophozoites were treated with 600 nM 17-AAG for 24 h. 0.2% DMSO was used as control. Treated cells were then induced for encystation in LG medium. Cells were also treated with 200 μM DTT for 5 h to induce ER stress and treated cells were then induced for encystation.

### Immunoblot

Equal number of trophozoites and cyst were harvested and lysed in laemmli buffer. Lysate was resolved on 10% polyacrylamide SDS gel under reducing conditions and immunoblot for EiHsp90 was carried out as described previously (Singh et al., 2014). Ponceau profile was used as a representative of equal loading.

### RNA Extraction and RT PCR

RNA extraction was carried out using TriZol reagent (Thermo Fisher Scientific) according to manufacturer’s instructions. The concentration and purity of the RNA extracted were evaluated using the Nanodrop spectrophotometer (Thermo Scientific). 2 μg RNA of all samples was used to synthesize cDNA using Verso cDNA Synthesis kit (Thermo Fisher Scientific) according to manufacturer’s instructions.

Primers for Aha1c, Sgt1, and HOP were designed as follows: Aha1c Fwd: 5′-CCGAGAGATTGACTGCGTTG-3′, Aha1c Rev: 5′-GGCCATGTGTTAAACCTCCA-3′ (product size: 154 bp), Sgt1 Fwd: 5′-CGCAGTGAGTTTCAACGAGA-3′, Sgt1 rev: 5′-TGTTAACAGCGTCCCAGTCT-3′ (product size: 242 bp), and HOP fwd: 5′-TAGAGCCGGACAATGAAGCA-3′, HOP Rev: 5′-ACGCCATCAAAGCTTCAGTG-3′ (Product size: 237 bp). rRNA primers were used as described before (Makioka et al., 2009). Amplification was performed in Mastercycler (Eppendorf) for 25 cycles. RT-PCR products were analyzed on a 2% agarose gel with ethidium bromide and rRNA amplicon was used as loading control for comparing trophozoites and cysts samples.

## Results

### Hsp90 Inhibition by 17-AAG Interferes with Erythrophagocytosis in *Entamoeba*

**FIGURE 1:**
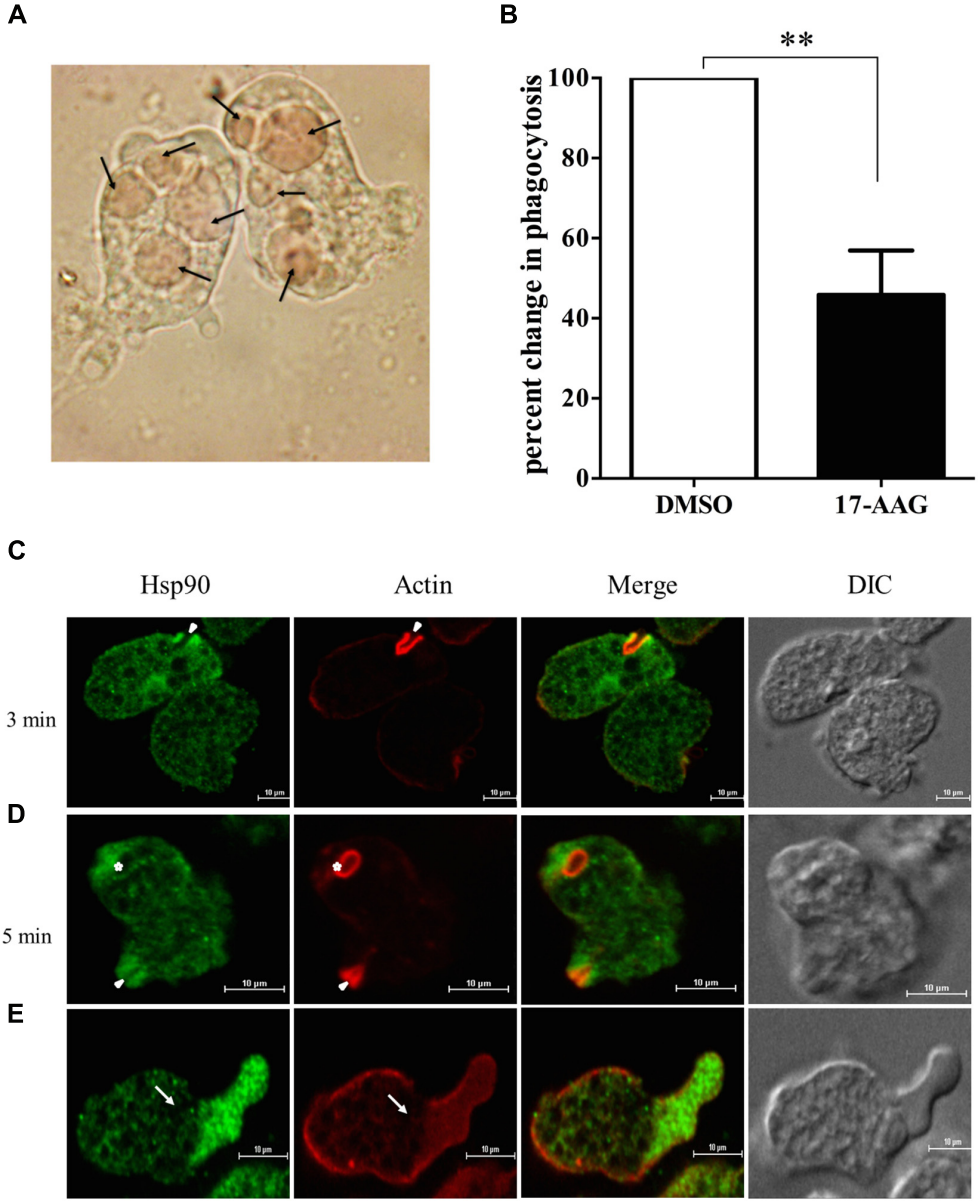
**Hsp90 regulates phagocytosis in *Entamoeba histolytica***. **(A)** Microscopic image of *Entamoeba* showing engulfed RBCs. Arrows show engulfed RBCs **(B)** Hsp90 inhibition by 600 nM 17-AAG decreases phagocytosis by 60% in comparison to DMSO treated cells (*P* < 0.01) Graph is representative of three experiments. **(C)** Localization of Hsp90 during erythrophagocytosis: *E. histolytica* cells were incubated with RBCs for different time points at 37°C. The cells were then fixed, stained with rabbit anti-Hsp90 and followed by secondary labeling with Alexa 488 (EhHsp90) and TRITC-Phalloidin (Actin) and Solid arrow represents phagocytic cups and asterisk indicates mature phagosome **(D)**. **(E)** Arrow represents pseudopod extension and enrichment of Hsp90. Differential interference contrast (DIC).

Erythrophagocytosis is a well-accepted method to quantitate phagocytosis rate by *Entamoeba. E. histolytica* cells were treated with 600 nM 17-AAG for 24 h. DMSO was used as control. Treated cells were then incubated with washed RBCs for 1 h. Microscopic observation was carried to ensure phagocytosis was optimum in control cells at end of 1 h (**Figure 1A**). Phagocytosis of control and treated cells was compared by absorbance of hemoglobin released from engulfed RBCs. It was observed that upon inhibition of Hsp90 by 17-AAG, there was a 60% decrease in phagocytosis by *Entamoeba* compared to DMSO treated cells (**Figure 1B**). This suggests Hsp90 plays a crucial role in regulation of phagocytosis.

In order to understand the role of Hsp90 in erythrophagocytosis, immuno-fluorescence staining was carried out. Hsp90 is found at the phagocytic cups along with actin during erythrophagocytosis (**Figure 1C**). Quantitation of the staining in images during erythrophagocytosis showed that fluorescence intensity of Hsp90 is higher in phagocytic cups compared to the rest of the cell and that there is a high degree of co-localization between Hsp90 and actin (Pearson correlation coefficient 0.8) implicating that Hsp90 is enriched in the cups along with actin. We also observed that Hsp90 did not get localized in mature phagosomes (**Figure 1D**).

Cell motility is required for host cell invasion and survival of *E. histolytica. Entamoeba* cells extend finger like projections called pseudopodia during cell movement that is driven by actin polymerization underneath the plasma membrane (Voigt and Guillen, 1999). It is observed that Hsp90 also gets accumulated at the leading edge of the cell during pseudopod extension and cell motility (**Figure 1E**). The enrichment of Hsp90 at the advancing pseudopods and phagocytic cups suggests that it may play a role in cell motility and initiation of phagocytosis.

### Hsp90 Expression Levels are Modulated during Encystation Process

**FIGURE 2:**
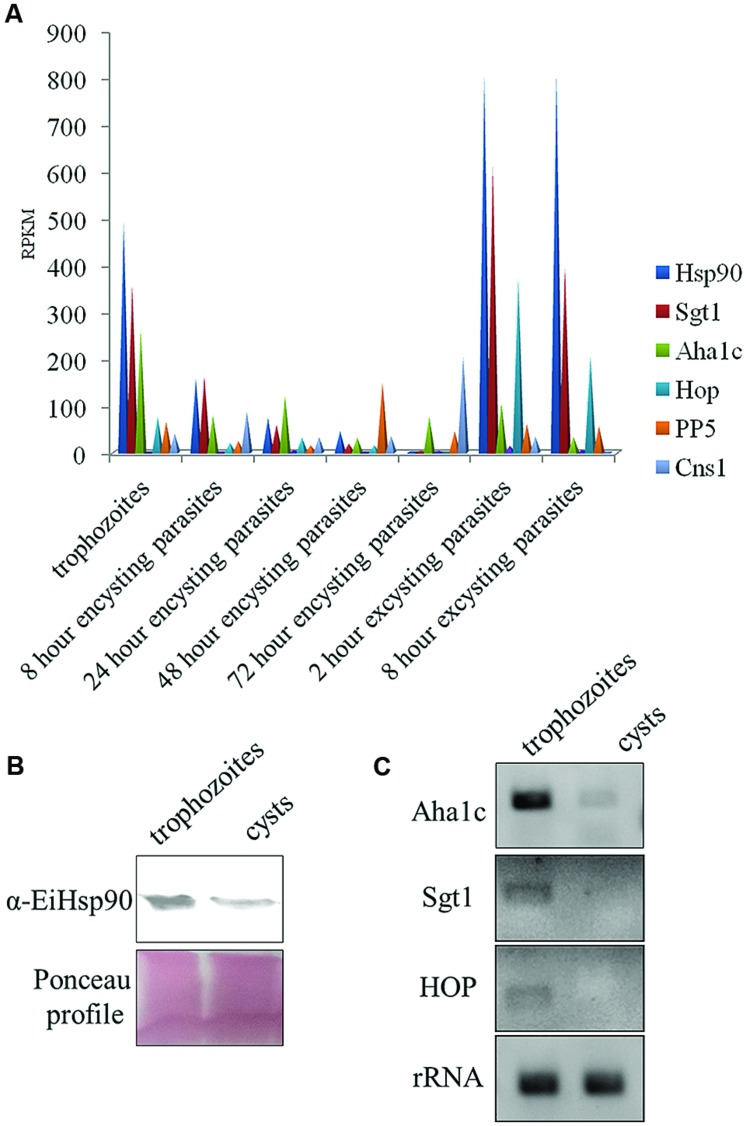
**Expression levels of Hsp90 and co-chaperones during *Entamoeba* life cycle**. **(A)** According to transcript data available at amoebadb.org, Hsp90 level decreases during encystation being least in cyst and again during excystation, the level of Hsp90 increases. Transcript profiles of co-chaperones Sgt1, Aha1c, Hop follow the same trend. However, transcripts for PP5 and Cns1 go up in 48 and 72 h encysting parasites (data adapted from amoebadb.org). **(B)** Validation of expression of Hsp90 at protein level by immunoblot at trophozoite and cyst stage. Ponceau profile is representative of equal loading. There is a marked decrease in expression level of Hsp90 in cyst. **(C)** Validation of expression levels of co-chaperones Aha1c, Sgt1, and Hop by semi- quantitative RT-PCR in trophozoites and cysts. rRNA was used as loading control. There is a significant decrease in transcript levels of Aha1c in cysts. Transcripts for Sgt1 and Hop were not detectable in cysts.

Hsp90 is known to regulate stage transition in various parasites and its levels get modulated in different stages of parasite life cycle. Hsp90 as a chaperone modulates both activation and inactivation of various client proteins. Some of the Hsp90 clients including transcription factors are activated upon a depletion of free Hsp90 pool and on the other hand many clients which are dependent on Hsp90 for their activation are degraded upon Hsp90 inhibition. In *Entamoeba*, very little is known about Hsp90 biology or the clients. Therefore, it is of interest to understand how Hsp90 levels are modulated in the entire life cycle. Hsp90 and its co-chaperone homologs were identified in *E. invadens* by homology search. Hsp90 (EIN_ 134370) co-chaperones identified included Aha1c (EIN_036190), Sgt1 (EIN_038830), Hop (EIN_109220), PP5 (EIN_054040), and Cns1 (EIN_168820). Transcript data for expression of these genes was retrieved from amoebadb.org (De Cádiz et al., 2013; Ehrenkaufer et al., 2013). It was observed that Hsp90 transcript levels decrease by 150-folds in cysts compared to trophozoites during encystation. On the other hand, during excystation Hsp90 levels were observed to increase again by 270-folds in excysting parasites compared to cysts (**Figure 2**). Hsp90 co-chaperones are known to regulate Hsp90 function by either activating or suppressing ATPase activity or by facilitating Hsp90-client interaction or aid in chaperone function. Therefore, expression levels of various Hsp90 co-chaperones were also analyzed. It was observed that with the exception of PP5 and Cns1, other three identified co-chaperones of Hsp90 in *Entamoeba* show expression profiles similar to Hsp90 (**Figure 2A**). Aha1c, an activator of Hsp90 ATPase activity, also shows a threefold decrease in transcript levels in cysts compared to trophozoites followed by an increase of 1.3-folds in excysting parasites.

Transcript data available at amoebadb.org was further validated by immunoblot of Hsp90 in trophozoites and cysts. A marked decrease in the expression of Hsp90 protein was observed in cysts (**Figure 2B**). Further, transcript levels of Hsp90 co-chaperones were also validated by semi-quantitative RT PCR. A significant down-regulation of Aha1c was observed in cysts and no transcript for Sgt1 and Hop was detected in cysts by RT PCR (**Figure 2C**). rRNA was used as control for equal loading. These observations altogether suggest that a functional Hsp90 multi-chaperone complex is involved in maintaining the trophozoite stage of parasite and a decrease in Hsp90 and co-chaperone levels co-relates with encystation.

### Hsp90 is a Negative Regulator of Encystation

**FIGURE 3:**
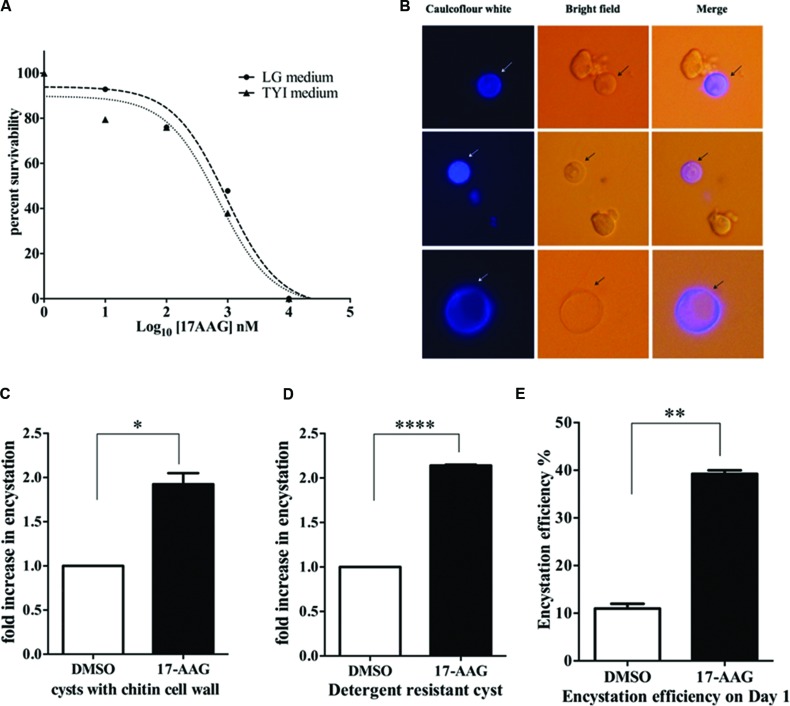
**Hsp90 is a negative regulator of encystation in *Entamoeba***. **(A)** Sensitivity of *E. invadens* Hsp90 inhibition using pharmacological inhibitor 17-AAG in both- growth medium TYI-S-33 and encystation medium (LG medium). The GI_50_ in TYI-S-33 and LG medium are 711 and 935 nM, respectively. **(B)** Cyst formation upon 72 h of encystation period in LG medium. Caulcoflour white stains the chitin cell wall of cysts. **(C,D)** Hsp90 inhibition using 17-AAG increases encystation efficiency by twofolds over control. Cyst formation was scored after 72 h encystation in LG medium by staining for chitin (^∗^*P* < 0.05) as well as by detergent resistant nature of cysts (^∗∗∗∗^*P* < 0.0001). Graph is representative of the mean of two biological replicates performed in triplicates **(E)** Hsp90 inhibition enhances time kinetics of encystation. In 24 h of encystation process in LG medium there is a threefold increase in number of cysts upon Hsp90 inhibition compared to control (^∗∗^*P* < 0.005). Graph is representative of the mean of two biological replicates.

Hsp90 is known to regulate stage differentiation in various other protozoa including *Giardia, Plasmodium, Leishmania*, and *Toxoplasma* (Wiesgigl and Clos, 2001; Graefe et al., 2002; Banumathy et al., 2003; Nageshan et al., 2014). Therefore, in light of the expression profile of Hsp90 in *Entamoeba* life cycle, its involvement in stage transition was examined. Firstly, we determined the GI_50_ value of Hsp90 inhibition for *E. invadens* by 17-AAG in both the growth medium TYI-S-33 and encystation LG medium. The GI_50_ in TYI-S-33 and LG medium are 711 and 935 nM, respectively, (**Figure 3A**). A sub lethal concentration of 600 nM was chosen for all further inhibition studies. *E. invadens* encystation was established *in vitro* and cyst formation was scored by either caulcoflour white staining of chitin (**Figure 3B**) or by counting detergent resistant cysts.

Trophozoites were treated for 24 h with 600 nM 17-AAG and DMSO was used as vehicle control. Post 24 h, 17-AAG and DMSO treated trophozoites were transferred to LG medium to induce encystation. Cyst formation was scored after 72 h. A twofold increase in cyst formation was observed in 17-AAG treated parasites compared to DMSO treated parasites (**Figures 3C,D**). We observed this increase is specific to Hsp90 inhibition and not a general stress by treating *E. invadens* with DTT to induce ER stress and score for encystation rate. No significant change was observed in encystation efficiency upon ER stress (Supplementary Figure S1). Further, it was observed that the conversion of 17-AAG treated trophozoites to cysts on day 1 of encystation itself was threefolds higher than DMSO treated cells (**Figure 3E**). This suggests that Hsp90 acts as a negative regulator of encystation process. Inhibition of Hsp90 promotes encystation.

## Discussion

Phagocytosis is a key hallmark of *Entamoeba* virulence. During invasion of intestine and extra-intestinal tissue, parasite has to phagocytize host epithelial cells and erythrocytes. Phagocytosis is an important bioprocess for virulence, growth and survival of *Entamoeba* (Bracha and Mirelman, 1984). There are extensive studies on signaling involved in phagocytosis (Sahoo et al., 2004; Aslam et al., 2012; Mansuri et al., 2014). Hsp90 has been also implicated in regulating cell motility and actin polymerization in cancer cells (Taiyab and Rao Ch, 2011). We demonstrate that inhibition of Hsp90 interferes with phagocytosis of RBCs by the parasite, which is important for parasite virulence and survival. We see specific localization of Hsp90 at the phagocytic cup and not in mature phagosome suggesting Hsp90 may play an important role for initiation of phagocytosis. We also see enhanced localization of Hsp90 in pseudopodia suggesting involvement of Hsp90 in cell motility.

We also studied the role of Hsp90 in *Entamoeba* life cycle during stage transition from trophozoites to cysts. As mentioned before, Hsp90 is known to regulate life cycle in many related protozoa like *Plasmodium, Giardia, Leishmania, Toxoplasma* etc. (Wiesgigl and Clos, 2001; Graefe et al., 2002; Banumathy et al., 2003; Echeverria et al., 2005; Nageshan et al., 2014; Rochani et al., 2014). Hsp90 is also known to regulate morphogenesis to filamentous form in *Candida albicans* (Shapiro et al., 2009).

In *Entamoeba*, encystation and excystation are complex biological processes. High throughput studies have determined the transcriptional and metabolite level changes during encystation and excystation (Jeelani et al., 2012; De Cádiz et al., 2013; Ehrenkaufer et al., 2013). There is an up-regulation of transporters, cytoskeletal proteins, proteins involved in vesicular trafficking, cysteine proteases, components of the proteasome, and enzymes for chitin biosynthesis and a down-regulation of metabolic genes. In this study, we have demonstrated for the first time involvement of Hsp90 in regulation of encystation process in *Entamoeba*. We show that upon Hsp90 inhibition there is a twofold increase in conversion of trophozoites to cysts. In a closely related parasite *Giardia lamblia*, Hsp90 has been shown to be a negative regulator of encystation (Nageshan et al., 2014). Hsp90 is traditionally well known to regulate various signaling and stress response pathways in higher eukaryotes. Cues for encystation are unknown but it is believed that environmental factors, like nutritional stress, change in gut micro-flora and host response are perceived by the parasite (Bailey and Rengypian, 1980; Vázquezdelara-Cisneros and Arroyo-Begovich, 1984; Byers et al., 2005). These cues act as a trigger for encystation. Hsp90 could possibly act as a transducer of these environmental cues leading to the initiation of encystation. Our study shows that inhibition of Hsp90 results in accelerated initiation of encystation process with a substantial increase in cysts observed on 24 h of encystation. We also examined if increased encystation is specific to Hsp90 inhibition or a general stress response. In *Plasmodium falciparum*, ER stress is known to induce stage transition to gametocyte (Chaubey et al., 2014). We induced ER stress at trophozoite stage by DTT treatment followed by induction of encystation. However, no significant change in encystation rate was observed upon ER stress, thus suggesting increased encystation is not a general stress response.

Transcriptome data suggests, that the level of Hsp90 transcript during excystation goes up by 270-folds compared to cysts (De Cádiz et al., 2013; Ehrenkaufer et al., 2013). Therefore, it will not be far-fetched to hypothesize that inhibition of Hsp90 during excystation can inhibit the excystation process. Further studies are required to understand the involvement of Hsp90 in excystation process. Hsp90 clients include many kinases, transcription factors and cytoskeletal proteins to name a few. Identification of Hsp90 clients in *Entamoeba* and their modulation in the encystation and excystation process will help in understanding the molecular details of Hsp90’s role in *Entamoeba* life cycle.

Our previous observation that Hsp90 is essential for survival of *Entamoeba* (Singh et al., 2014) and its involvement in regulation of phagocytosis, encystation and possibly in excystation indicates Hsp90 to be an excellent therapeutic target. Hsp90 is also known to regulate drug resistance in many fungal species (Rutherford and Lindquist, 1998; Cowen et al., 2009). Therefore, it will also be worth exploring a combination therapy with Hsp90 inhibitors in the cases of drug resistant amoebiasis.

## Conflict of Interest Statement

The authors declare that the research was conducted in the absence of any commercial or financial relationships that could be construed as a potential conflict of interest.
