# Supplementary material for: Heat Shock Protein 90 regulates encystation in Entamoeba
Source: Front Microbiol. 2015 Oct 13;6:1125. doi: 10.3389/fmicb.2015.01125 (PMC4602144; doi:10.3389/fmicb.2015.01125)

**Heat Shock Protein 90 regulates encystation in *Entamoeba***

**Meetali Singh^1^, Shalini Sharma^2^, Alok Bhattacharya^2^ and Utpal Tatu^1*^**

^1^Department of Biochemistry, Indian Institute of Science, Bangalore – 560012, India

^2^School of Life Sciences, Jawaharlal Nehru University, New Delhi-110067, India.

***Correspondence:** Utpal Tatu, Department of Biochemistry, Indian Institute of Science, Bangalore – 560012, India. Email: [tatu@biochem.iisc.ernet.in](mailto:tatu@biochem.iisc.ernet.in)

**Supplementary data**

**Fig S1**: **Effect of DTT treatment on encystation.** No significant difference in encystation rate was observed upon ER stress induced by treatment with 200 μM DTT.


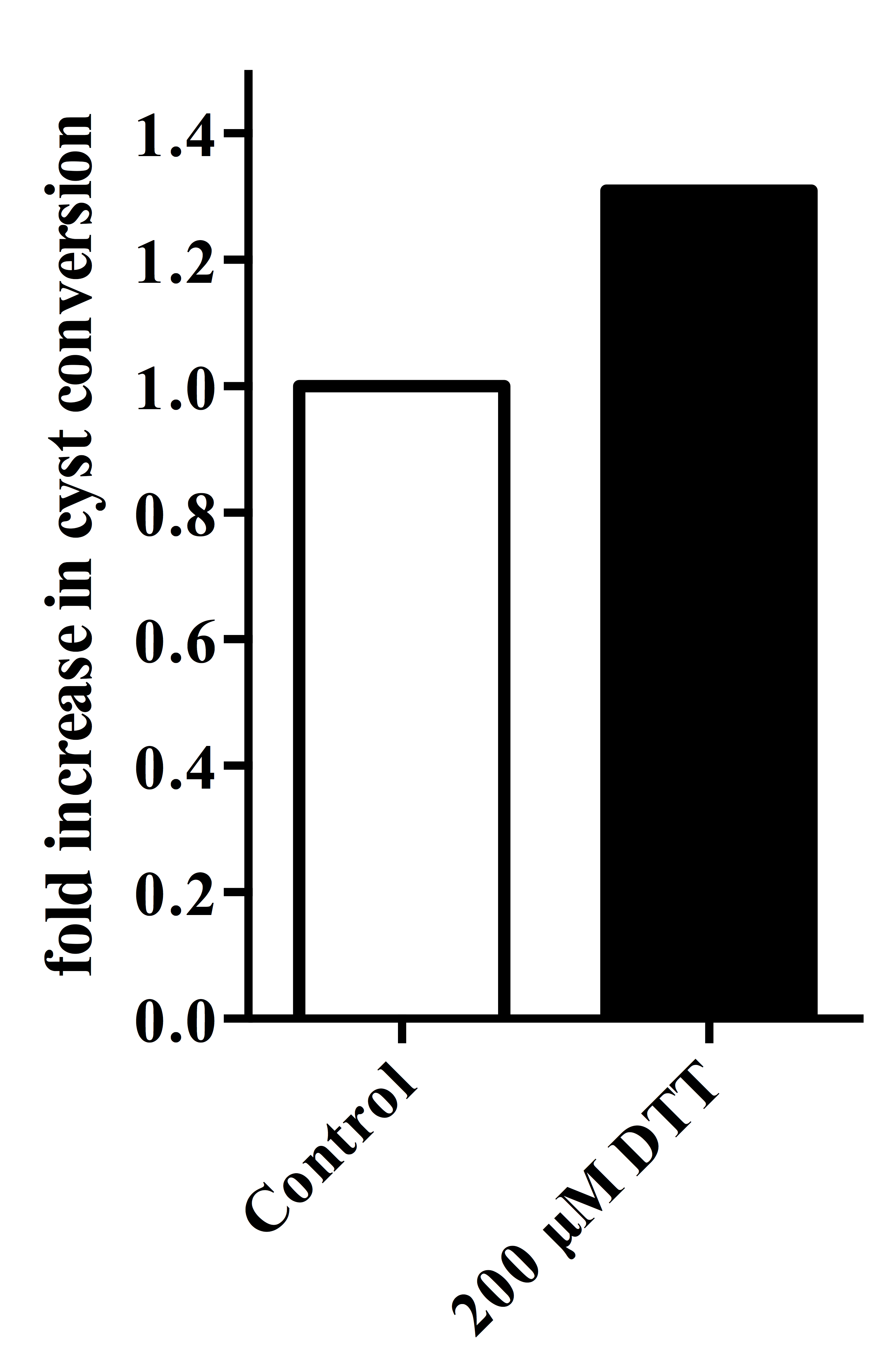

Supplement: Supplementary file 1 [file Data_Sheet_1.DOCX]
